# Remarks on Tar-Vapour, as a Remedy in Diseases of the Lungs. Illustrated with Cases Treated at the General Military Hospital, Fort Pitt

**Published:** 1822-10

**Authors:** James Forbes

**Affiliations:** Deputy Inspector of Hospitals.


THE LONDON
### Medical and Physical Journal.

4 OF VOL. XLVIII.] OCTOBER, 1822. [No. 284.

For many fortunate discoveries in medicine, and for the detection of numerous errors, the world is indebted to the rapid circulation of Monthly Journals; and there never existed any work, to which the Faculty, in Europe and America, were under deeper obligations, than to the Medical and Physical Journal of London, now forming a long, but an invaluable, series.—RUSH.

### ORIGINAL COMMUNICATIONS,

### SELECT OBSERVATIONS, &c.

ART. I.—*Remarks on Tar-Vapour, as a Remedy in Diseases of the Lungs. Illustrated with Cases treated at the General Military Hospital, Fort Pitt.* By JAMES FORBES, M.D. Deputy Inspector of Hospitals.

FROM the numerous, and apparently satisfactory, cases brought forward by Sir A. Crighton and others, in support of the efficacy of the vapour of tar in the cure of pulmonary phthisis, it was natural to form somewhat sanguine anticipations of its success; for, however sceptical we may have become on the powers of any remedy, or class of remedies, in producing any thing beyond a partial alleviation or temporary retardation of the sometimes slow, but always sure, march of this formidable disease, yet it was possible that a remedy had at length been found, which would alleviate the sufferings, retard the progress, and perhaps cure, a malady which had hitherto resisted every means; although the examination, *post mortem*, of ninety patients who have sunk under the disease within the last eighteen months, by no means prepared us to consider this as a probable occurrence. The discoverers of new remedies are ever prone to exaggerate their good effects, and to impose on their brethren by opinions too hastily and hypothetically deduced; yet, of the correctness or truth of Sir A. Crighton's statement, we can entertain no doubt; but, from what has taken place at this hospital during the trial of the remedy, I am inclined to believe that the cases which he has recorded as having been cured or improved by the tar-vapour were not cases of ulcerated lungs, but were more nearly allied to chronic catarrhal affections, between which and tubercular phthisis it is often difficult, sometimes impossible, to draw a distinction.

The manner in which the remedy has been exhibited in this hospital, is as follows:—"In a small ward, containing six patients, are placed two common metal pots, into each of which two pounds of tar are poured, with the addition of one ounce of the supercarbonas potassæ, in order to absorb the pyroligneous acid. Under these vessels is placed a spermaceti-oil lamp, by the heat of which the vapour is disengaged. The windows and door of the ward are closed, to prevent the escape of the vapour. Hitherto we have generally used it six hours daily, but it may be used for a much longer period. Guided by Sir A. Crighton's opinion, that most advantage appeared to be afforded by the remedy to patients affected with tubercular consumption, six cases of that description were selected. From the manifest bad effects of the remedy in *all* these cases, we were obliged shortly to relinquish its use; and, not only in them, but in all cases of a like nature. In general, it rendered the breathing more difficult, and cough almost incessant; the expectoration was diminished, the pulse commonly accelerated, and anxiety and restlessness produced. Its effects will be better shown from the two following cases.

Case I.—Simon Burton, æt. 23, of strumous diathesis, has laboured under pectoral complaints for nearly two years; breathing very much oppressed; cough frequent, with copious purulent expectoration; pulse 120, moderately full; perspires copiously at night, and his bowels are generally constipated; tongue clean; appetite pretty good. He is much emaciated; his symptoms undergo considerable exacerbation in the evening, and he is hectic to an extreme.

*April 29th.*—Ordered the tar-vapour.

*30th.*—Passed a restless night; breathing more laborious, and cough more troublesome.

*May* 1.—Respiration continues to become more difficult; cough undiminished; complains much of thirst; pulse 132.

*Three o'clock, same day.*—Since last report, his respiration has become extremely difficult; the cough almost incessant; and there is anxiety of countenance and general inquietude. Removed immediately to another ward. This patient, on his removal from the tar-vapour ward, became in every respect easier; respiration was performed with comparative freedom, and the cough and other symptoms suffered considerable abatement. I have little doubt that, had the use of the remedy been persisted in, he would shortly have died by suffocation. He died on the 21st of May, and the lungs were found much tuberculated: most of the tubercles in a state of ulceration, and strong adhesions between the pleuræ.

Case II.—William Taylor, æt. 22, has laboured under pulmonary affection for eleven months. Respiration hurried and laborious; cough frequent and severe, accompanied with purulent expectoration; pulse 120, of good strength and fulness; occasionally perspires profusely, and his bowels are prone to constipation. Emaciation and debility considerable; tongue clean; appetite indifferent. Towards evening, his breathing becomes more difficult, and the other symptoms also suffer considerable exacerbation. The pulsation of the heart is widely diffused, and is best felt at the epigastrium.

*April* 29.—Put under the use of the tar-vapour.

30*th*.—Had a restless night, and his breathing is rather more oppressed; the cough more distressing, and there is general inquietude; pulse 124.

*Three o'clock, same day.*—Respiration much more impeded, and the other symptoms considerably aggravated. He anxiously desires to be removed to another ward.

The tar-vapour obviously did harm in this case, as in the other. The patient died about eight days after his removal. He was often heard piteously to lament his ever having tried the "tar-smoke," as he had "never recovered the breath he had lost under its use."

On dissection, the lungs were found a complete mass of disease, much tuberculated, and containing many small vomicæ. The diffused pulsation of the heart led to the opinion that there existed some disease of that organ: after death, however, no morbid appearance of it was discovered. This is a circumstance worthy of notice, as such an appearance is calculated to deceive many. I believe it is frequently met with in emaciated subjects.

In the other four cases, the disease followed its usual progress, and, on dissection, they were all found to have tuberculated lungs. In one of them, a large vomica occupied the upper part of the left lung; in another, several small vomicæ were found. In these the tar-vapour did not produce such distressing symptoms, but yet their complaints were considerably aggravated; and so much so in two of them, that it became absolutely necessary to discontinue the remedy; and, in all these cases, the pulse became more frequent during its exhibition, the cough and dyspnœa more urgent, and the expectoration diminished, which last effect seldom failed to render the breathing more oppressed. One appeared at first to improve somewhat under its use; but latterly, and as his complaints became worse, (for they were never obviously retarded by its employment,) it certainly did manifest harm, producing the same distressing effects as in the cases detailed, which did not cease for several hours after exposure to the vapour. The quantity of expectoration was occasionally diminished by its use; and, though his symptoms were not at all times aggravated by this change, yet they generally were so, and he always experienced an alleviation of the dyspnœa, and was otherwise considerably benefited, by the return of a free and copious expectoration.

From the unsuccessful results of the cases above mentioned, I had nearly given up the hope of the remedy affording any advantage. I was inclined, however, to think that some of its injurious effects were to be attributed to some imperfection in the process by which the vapour was produced. After various trials, the mode already described was adopted; and since that no case has occurred in which the symptoms were so much aggravated as in those of Ruston and Taylor.

In not one case, out of nineteen submitted to its action, has the tar-vapour given evident proofs of its power either to arrest or to ameliorate the symptoms of the disease. It is true that several patients, during the two or three first days, have expressed themselves benefited by it; but these very patients have, in a short time after, either suffered so much from its effects as to request their removal to another ward, or have entirely denied their having received any benefit from it. It is well known that phthisical patients, in general, judge very erroneously respecting their complaints, and from day to day return the same answer, "I am better," when in fact they are daily getting worse. It may be observed, farther, that such patients not unfrequently over-rate the good effects of medicine, and are sometimes apt to bestow unbounded praise on the most inert remedies. It has often appeared to me, that whatever is capable of interrupting, or changing in any degree, the protracted and monotonous progress of phthisis, is to the patient a source of gratification. This I apprehend to have been the case with many patients admitted into the tar-vapour ward, who, perhaps, sanguine in their expectations of its doing them good, do not, for the first day, (during which it gives little inconvenience,) make any complaint, but perhaps give to it some praise. They, in a short time, however, either make loud complaints, or cease to allow that they have been benefited.

The most direct and obvious effect of tar-vapour is to diminish the quantity of expectoration. This, in catarrhal affections, is followed by no unpleasant symptoms: on the contrary, the cough and dyspnœa, but more especially the former, undergo a corresponding diminution; but in phthisis nearly the reverse of this happens, for, in proportion as the sputa become diminished, so does the cough become more frequent, and the difficulty of breathing greater. We have observed, however, that some phthisical patients may remain for a considerable time under the use of this remedy, without experiencing any unpleasant effects; but, in the generality of cases, it has produced the bad effects already mentioned. It has appeared that, so long as the tar-vapour does not suppress the expectoration in any considerable degree, it may be found, in some cases of phthisis, a grateful remedy; and we have reason to infer this from the circumstance of some patients, in a very advanced stage of the disease, expressing their partiality for it: but, unfortunately, this has only been observed during the first days of using it, for, in every instance in which it was continued for any considerable length of time, it has invariably occasioned a scanty and difficult expectoration.

In all cases where the disease was far advanced, the tar-vapour aggravated the symptoms, and coughing has taken place sooner or later, according to the greater or less extent of disorganization; and these effects have, in every case, appeared to arise from a diminution in the quantity of the expectoration. Patients with diseased lungs, as above mentioned, may remain for a considerable time under the remedy, with little or no inconvenience; but, as soon as any considerable deficiency takes place in the expectoration, the cough, which before may have been moderate, now becomes much more frequent and distressing; and, in most cases, this has been accompanied with increased difficulty of breathing. It may here be necessary to remark, that to an inattentive observer, and even to the patient himself, the expectoration in some cases would appear to be increased, or at least not diminished in quantity; but, when carefully examined, will be found to be much less purulent than before the use of the vapour,—to have assumed a frothy appearance,—and to contain a much larger proportion of mucus and saliva.

In one or two cases of incipient consumption, in which the tar-vapour was used, though its bad effects were not evident, yet no good apparently resulted from it. Indeed, I am sorry to say that we have not yet seen one unequivocal case of the disease in which it appeared to be of the smallest benefit, but several wherein it could hardly be said to have produced any bad effects, excepting slight head-ach and thirst; symptoms which most patients experience on commencing the use of the remedy.

What I understand by chronic catarrh is a disease succeeding to previous inflammatory action of the bronchiæ in particular, but which is itself unattended with any lesion or inflammation, but, on the contrary, has for its cause a morbid relaxation of the lining membrane of these tubes, by which it happens that a preternaturally increased secretion takes place: in other words, that this membrane takes on the same action in chronic catarrh, as the mucous lining of the urethra does in gleet. This disease I believe to be accompanied, in many cases, with a corresponding state of the body,—not as an effect, but independently exciting, and sometimes acting as a cause of the other; and I apprehend that the cough accompanying this complaint is no more than a necessary effect of a superabundant secretion of mucus. By the above I would be understood to have described a complaint most properly deserving the name of chronic catarrh, and that all other pectoral affections essentially differing from it are improperly so termed. I have only entered into this explanation with a view to observe, that the tar-vapour is likely to be found most beneficial in the complaint above described, and in all cases accompanied with the same weakened condition of the vessels. The tar-vapour appears to possess stimulant properties, and perhaps astringent ones: it is therefore peculiarly well adapted as a remedy to restore the energy of debilitated absorbing or secerning vessels. Upon no other principle can we explain its apparent good effects on chronic catarrh, than by supposing that its stimulant power excites a new and salutary action in the mucous excretory vessels of the bronchiæ; and the very circumstance of its proving hurtful in phthisis, because the lungs and sanguiferous system are in a high state of excitement, tends very much, in our opinion, to support this conclusion. I shall here subjoin some cases which have apparently been benefited by the remedy.

Case I.—Barnett Gromley, ætat. 35, complains of frequent cough, with which he expectorates a mucous matter in considerable quantity. Respiration oppressed on using exercise; pulse 100, moderately full and strong. On using any great exertion, he is occasionally troubled with palpitation of the heart. Some perspiration at night; bowels regular; tongue furred; appetite good. Within the last month his complaints have become less severe, and he has gained some flesh; at present he is not much debilitated, nor is his emaciation at all considerable. His complaint commenced about eighteen months ago in Jamaica. He is of a sanguineous habit, and well formed about the chest.

*June* 12.—Ordered the tar-vapour.

13*th*.—Passed a good night, and says the tar-vapour has relieved the difficulty of breathing, and diminished the frequency of the cough. Pulse as yesterday.

14*th*.—Says he has experienced considerable relief from the remedy. The cough is now comparatively trifling, and the dyspnœa is considerably diminished. Pulse as before.

15*th*.—Expresses himself nearly free from complaint. Pulse unchanged.

16*th*.—Says he has now no cough, and is much improved in other respects.

This patient continued to improve till the 24th, when he was transferred to the convalescent hospital free from all complaint, except some inconsiderable remains of dyspnœa.

Case II.—John Ring, ætat. 27, is affected with frequent cough, more especially towards morning; the expectoration is apparently mucous; pulse 100, moderately full and strong; breathing oppressed on using exercise, but when at rest is performed with little difficulty; tongue clean; appetite, &c. natural. He has fallen away a good deal in flesh and strength, but within the last six weeks his complaints have improved. He has laboured under them for a year and a half; they commenced in Jamaica. He is of a sanguineo-phlegmatic habit, and rather flat and narrow about the chest.

*June* 12*th.*—Put under the use of the tar-vapour.

13*th.*—Passed a good night, and his cough has been less troublesome. Pulse as yesterday.

14*th.*—His breathing is not nearly so much oppressed; the expectoration is considerably diminished, and the cough is comparatively trifling. Pulse 104.

16*th.*—The cough and expectoration are now inconsiderable. Pulse 100.

17*th.*—Says he is free from cough, and nearly so from dyspnœa. Pulse as yesterday.

18*th.*—Dyspnœa inconsiderable; pulse 100. This patient was transferred on the 24th to the convalescent hospital, free from complaint.

Case III.—Thomas Perkins, 1st Foot, ætat. 28, complains of frequent troublesome cough, with copious purulent expectoration. Respiration hurried and oppressed on using exercise, but when at rest appears to breathe with freedom; pulse 92, rather small, but of good strength; appetite indifferent; bowels loose, with some degree of tenesmus; emaciation and debility considerable; general surface shrunk and dry, and he complains of cold; tongue slightly white, with some thirst; says that he feels a soreness in his chest, but has no degree of pain. He has laboured under the above complaints for two years; they commenced in India, in which climate he remained for nine years. States that, about six years ago, he was attacked with dysentery; the present complaint of the bowels came on about two months ago, and has continued without any remission. Astringent mixture to check the diarrhœa, and a common pectoral mixture for his cough, were prescribed. On the following day the purging was diminished, but his breathing more difficult, cough more troublesome, and the pulse increased to 104. The same treatment was continued, and the following day he expressed himself considerably relieved in the chest by the tar-vapour; his breathing less oppressed, cough and expectoration much diminished. On the fifth day after his admission (June 11th,) the expectoration was observed to have got the purulent, and more of the mucous appearance; his breathing was performed with more freedom; cough continued to decrease; pulse 104; yesterday it was 124.

This pectoral affection continued improving daily, but on the 15th of June the diarrhœa was increased, when the power of the astringent mixture was augmented by the addition of the extract hœmatox. From this date he continued improving in all his symptoms until seven o'clock P.M. of the 21st June, when he was attacked with the following symptoms:—He was suddenly deprived of motion, accompanied with irregular actions of the muscles. Two hours afterwards the body appeared paralyzed, and he complained of a sense of weight in the forehead; his pupils were dilated and impenetrable to light; lips and face livid; breathing hurried, but free from stertor; pulse 130, moderately full and strong. When interrogated, answers distinctly, but with reluctance. Twenty-six ounces of blood were now taken from his arm, and sixteen ounces by cupping from the temples. Next day he said that he had quite recovered the power of motion, but still complained of the weight in the forehead. Eyes natural; pulse 104, moderately full; bowels open. Fourteen ounces again taken by cupping on the temples. In the evening the sense of weight was gone, but his pupils were considerably dilated. His head was now shaved, and a large blister applied to it.

*June 23d.*—Pupils natural in size and sensible to light; but he experienced a sense of coldness and numbness in his inferior extremities, and was unable to support his body on them. Pulse 120, small. Blister did not rise well, and another was applied to the neck.

*24th.*—Had recovered the power of motion, and the numbness of the legs was gone. Face pale; lips livid; pulse 110; cough and dyspnœa much relieved.

*25th.*—The livid colour of the lips disappeared, and his only complaint debility. Pulse 96. He continued daily to improve under the influence of the tar-vapour, and was discharged from the hospital, 20th November, greatly improved.

| Diseases. | Total treated. | Cured. | Improved. | No effect. | Bad effects. |
|---|---|---|---|---|---|
| Phthisis Pulmonalis | 19 | None. | None. | 8 | 11 |
| Catarrhus Chronicus | 32 | 8 | 6 | 18 | None. |

